# Comparison of Intracervical Foley’s Catheter With Vaginal Misoprostol Versus Intravaginal Misoprostol Alone for Cervical Ripening and Induction of Labor

**DOI:** 10.7759/cureus.44772

**Published:** 2023-09-06

**Authors:** Nivedita A Kadu, Shobha Shiragur

**Affiliations:** 1 Department of Obstetrics and Gynaecology, Shri B.M. Patil Medical College, Hospital and Research Institute, Vijayapura, IND

**Keywords:** misoprostol, bishop score, cervical ripening, meconium stained amniotic fluid, foley's catheter, augmentation, induction of labour

## Abstract

Introduction

Induction of labor implies stimulation of contractions before the spontaneous onset of labor, with or without membranes. Augmentation refers to the enhancement of spontaneous contractions that are considered inadequate because of failed cervical and fetal descent. This study compared the effectiveness of intracervical Foley catheter insertion and vaginal misoprostol versus only vaginal misoprostol in the induction of labor and other outcomes relted to it.

Methods

The present study was a randomized controlled trial that included 148 women divided into two groups: (i) Group A, which received intracervical Foley catheter insertion and vaginal misoprostol (25 µg), and (ii) Group B, which received intravaginal administration of tablet misoprostol (25 µg) alone. We compared the median time from the time of induction to vaginal delivery, incidence of cesarean delivery, chorioamnionitis, puerperal infection, uterine tachysystole, neonatal information at delivery, and discharge status (i.e., birth weight, neonatal intensive care unit (NICU) admission, and neonatal death) between groups.

Results

We found that the rates of puerperal infection (n=36; 48.6%) and meconium-stained amniotic fluid (n=45; 60.8%) were higher in Group B than in Group A (n=20; 27.0% and n=25; 33.8%, respectively), which were statistically significant differences (p=0.0066 and p=0.0009, respectively). In addition, NICU admission was higher in Group B (n=47; 63.5%) than in Group A (n=30; 40.5%), which was a statistically significant difference (p=0.0051).

Conclusion

An intracervical Foley catheter with 25 µg of misoprostol was more effective for induction of labor than 25 µg of intravaginal misoprostol alone every six hours for a maximum of four doses in terms of induction to delivery interval, meconium-stained amniotic fluid, mode of delivery, intrapartum complications, and puerperal infection.

## Introduction

Induction of labor (IOL) is defined as the stimulation of contractions before the commencement of spontaneous labor, with or without ruptured membranes. When the cervix is closed and uneffaced, labor induction will frequently start with cervical ripening, a procedure that typically uses prostaglandins to soften and open the cervix [1,2]. Augmentation describes the improvement of spontaneous contractions that are deemed insufficient due to unsuccessful cervical ripening and fetal descent. IOL, i.e. starting labor before the spontaneous onset of labor in a viable pregnancy, is considered when there is a high risk associated with continuing the pregnancy or at the request of the pregnant woman at term. IOL is becoming more prevalent in contemporary obstetrics and provides better care for the mother’s fetus [3,4]. According to the World Health Organization’s (WHO) Global Survey on Maternal and Perinatal Health, which included 24 countries, IOL was used in 9.6% of all deliveries in 2006, and 22.5% of those in the United States in the same year [5].

Cervical ripening can be accomplished by two types of methods, mechanical or pharmacological. Mechanical methods involve one of the following: the insertion of a catheter with a balloon insufflation through the cervix into the extra-amniotic space; the placement of laminaria tents or their synthetic equivalent (e.g., Dilapan-S®, Medicem Group a.s., Czech Republic) into the cervical canal; or the injection of fluid by insertion of a catheter into the extra-amniotic space [6,7]. Pharmacological methods include the use of many different agents such as prostaglandin (PG) (PGE2 or PGE1), progesterone receptor antagonists (mifepristone), oxytocin, and nitric oxide (NO) donors, but the most commonly used agents are PGs and oxytocin. 

Misoprostol, a synthetic PGE1 analog, is often utilized because it is inexpensive and simple to preserve (refrigeration is not required), despite the fact that it has not been approved for IOL [8,9]. Cervical softening is accomplished through the breakdown and dissolution of extracellular collagen. Misoprostol can be administered by various methods, such as orally, buccally, sublingually, vaginally, and rectally [10,11]. Intravaginal administration provides a more gradual start and higher bioavailability than oral or sublingual routes, causing a sudden rise in plasma misoprostol concentration, which is better for managing postpartum hemorrhage (PPH).

With vaginal delivery occurring in 73% of pregnancies and hyperstimulation syndrome occurring in 3.6% of women, the first doses of vaginal misoprostol used to be 50 μg every two hours, with a maximum dose of 600 μg [9,10]. Since then, smaller dosages have been advocated for IOL, to lessen the side effects of misoprostol [6,11]. After multiple research projects were conducted, WHO and the International Federation of Gynecology and Obstetrics (FIGO) approved a vaginal misoprostol dosage of 25 μg every four hours for a maximum of six doses [12,13]. Before 2018, no studies on the use of sublingual misoprostol for IOL with a viable pregnancy had been published. However, a pharmacokinetics study of misoprostol taken by multiple routes revealed that the sublingual route had higher bioavailability than the vaginal route. Therefore, in this study, we aim to compare intracervical Foley catheters with vaginal misoprostol versus vaginal misoprostol alone for cervical ripening and IOL.

## Materials and methods

Method of data collection

We conducted this study at the Department of Obstetrics and Gynecology of Shri B.M. Patil Medical College, Hospital and Research Center, Vijayapura, Karnataka, India. Patients fulfilling the inclusion criteria described below were included in the study. The Institutional Ethics Committee, Shri B.M. Patil Medical College, Hospital and Research Center approved the study (approval number: IEC/No-09/2021), and we obtained informed consent from all patients included in the study.

The inclusion criteria were: a singleton pregnancy of gestational age of 37 weeks or more, a live fetus with a cephalic presentation, first and second gravida, non-severe pre-eclampsia, cervix <2 cm dilated, and age of mother between 18 and 35 years. The exclusion criteria were: premature rupture of membranes, prior uterine incision, fetal anomalies, antepartum hemorrhage, multiple pregnancies, major-degree cephalopelvic disproportion, malpresentation, maternal medical disorder (e.g., heart disease or asthma), and severe pre-eclampsia or eclampsia. The total sample size was 148 women.

Methodology

On admission to the labor room, we obtained consent from each patient to participate in the study and ordered relevant investigations. A complete history was taken, a general physical examination was done, and labor was monitored according to the modified WHO partograph.

The participants were divided into two groups: (i) Group A, receiving intracervical Foley catheter insertion and vaginal misoprostol (25 µg), and (ii) Group B, receiving intravaginal administration of tablet misoprostol (25 µg) alone. For Group A, a 16F Foley catheter was inserted through the internal os of the cervix under aseptic conditions. The balloon was inflated with 30 mL of distilled water, and the catheter was strapped in the middle of the thigh with elastic tape without tension to be left for 24 hours unless expelled. Per vaginal examination was done for the progression of Bishop’s score and further depended on oxytocin augmentation or artificial rupture of the amniotic membranes (ARM). The women in Group B received a tablet of misoprostol (25 µg) in the posterior fornix every six hours for 24 hours (maximum of four doses). At the end of 24 hours, per vaginal examination was done for the progression of Bishop’s score and further depended on oxytocin augmentation or ARM. The titrating dose of oxytocin of 0.5-1 mUnit/minute IV, titration 1-2 mUnit/minute within 15-60 minutes until the contraction pattern is reached (usually 6 mUnit/min); dose may be decreased after the desired frequency of contractions is reached and labor has progressed to 5-6 cm dilation.

We determined the following information after analyzing the patient data: the median time from the time of induction to vaginal delivery, the proportion of patients who delivered vaginally within 24 hours, the proportion of patients who delivered vaginally within 48 hours, the incidence of cesarean delivery rates in each age group, chorioamnionitis, puerperal infection, uterine tachysystole, neonatal information at delivery, and discharge status (i.e., birth weight, NICU admission, and neonatal death).

Statistical analysis

IBM SPSS Statistics for Windows, Version 20.0 (Released 2011; IBM Corp., Armonk, New York, United States) to perform a statistical analysis of the data after they were entered into a Microsoft Excel spreadsheet (Microsoft Corporation, Redmond, Washington, United States). We used independent t-tests to compare continuous variables that were regularly distributed across two groups. We applied the Mann-Whitney U test for variables that were not normally distributed. We used the chi-square test to compare categorical variables across two groups. Differences were judged to be statistically significant at p ≤ 0.05. All statistical analyses were run in a two-tailed fashion.

## Results

**Figure 1:**
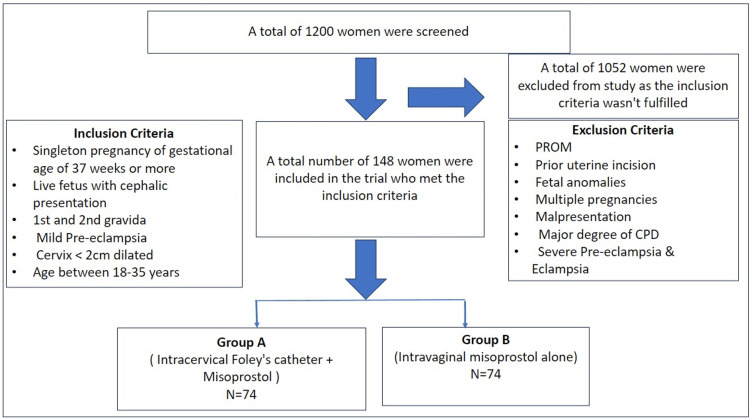
Flow chart of the study PROM: premature rupture of membranes

A total of 1200 women were screened, of which 148 met the inclusion criteria and were included in the study. They were then randomly allocated to either Group A (n=74) or Group B (n=74), as shown in Figure 1. The demographic characteristics of both groups are shown in Table 1.

**Table 1:** Demographic characteristics

	Group A (Foleys catheter + Misoprostol), n=74	Group B (Misoprostol), n=74	p-value
Age (years), mean ± SD	25.29 ± 4.1	25.37 ± 4.1	0.86
Primigravida, n (%)	38 (51.4 %)	49 (66.2 %)	0.05
Multigravida, n (%)	36 (48.6 %)	25 (33.8 %)	0.05
Gestational age (weeks), mean ± SD	39.26 ± 1.0	39.28 ± 1.13	0.92
Bishop’s score, mean ± SD	2.9 ± 0.7	2.8 ± 0.7	0.51

In Group A, the time interval from induction of labor to delivery was 5.893 ± 1.415 hours, whereas that in Group B was 7.284 ± 1.36 hours, and this difference was statistically significant (p<0.001). In Group A, 28 (37.8%) patients underwent lower (uterine) segment Caesarean section (LSCS), and 46 (62.2%) patients underwent vaginal delivery, whereas in Group B, 29 (39.2%) patients underwent LSCS, and 45 (60.8%) patients underwent vaginal delivery, and these differences were not statistically significant (p=0.50). The differences in the rates of puerperal infection (p=0.005) and intrapartum complications (p=0.016) were statistically significant between groups, whereas that of atonic PPH (p=0.24) was not statistically significant (Table 2). 

**Table 2:** Maternal outcomes PPH: postpartum hemorrhage

	Group A (Foleys catheter + Misoprostol), n=74	Group B (Misoprostol), n=74	p-value
Induction to delivery time (hours), mean ±SD	5.89 ± 1.41	7.28 ± 1.36	< 0.001
Vaginal delivery, n (%)	46 (62.2%)	45 (60.8%)	0.50
Cesarean delivery, n (%)	28 (37.8%)	29 (39.2%)	0.50
Puerperal infection, n (%)	20 (27%)	36 (48.6%)	0.005
Intrapartum complications, n (%)	1 (1.4%)	10 (13.5%)	0.016
Atonic PPH, n (%)	5 (6.8%)	2 (2.7%)	0.24

The indications for cesarean deliveries were comparable between groups. In Group A, the indication for LSCS was failed induction in eight (10.8%) patients, failure to progress with cephalopelvic disproportion (CPD) in six (8.1%) patients, and fetal distress in 14 (18.9%) patients. In Group B, the indication for LSCS was failed induction in three (4.1%) patients, failure to progress with CPD in 14 (18.9%) patients, and fetal distress in 12 (16.2%) patients. These differences in indications for LSCS between groups were statistically significant (p<0.0001) (Table 3).

**Table 3:** Indication for cesarean deliveries CPD: cephalopelvic disproportion

	Group A (Foleys catheter + Misoprostol), n=74	Group B (Misoprostol), n=74	p-value
Failed induction, n (%)	8 (10.8 %)	3 (4.1 %)	< 0.001
Failure to progress with CPD, n (%)	6 (8.1 %)	14 (18.9 %)	
Fetal distress, n (%)	14 (18.9 %)	12 (16.2 %)	

Statistically significant differences in the rates of NICU admission and meconium-stained amniotic fluid were found between both groups (Table 4). In Group A, the mean birth weight of babies was 2.7257±0.1664 kg, whereas in Group B, it was 2.7649±0.1968 kg. The distribution of mean birth weight within the group was not statistically significant (p=0.1930) (Table 4).

**Table 4:** Neonatal outcome NICU: neonatal intensive care unit

	Group A (Foleys catheter + Misoprostol), n=74	Group B (Misoprostol), n=74	p-value
Birth weight (Kg), mean±SD	2.72 ± 0.16	2.76 ± 0.19	0.193
NICU admission, n (%)	30 (40.5 %)	47 (63.5 %)	0.005
Meconium stained amniotic fluid, n (%)	25 (33.8 %)	45 (60.8 %)	0.0009

## Discussion

In this study, both intracervical Foley catheter + misoprostol (Group A) and intravaginal misoprostol alone (Group B) were used for IOL and cervical ripening. The results showed that the mean induction to delivery time was significantly shorter (5.8±1.41 h) within Group A as compared to Group B (7.2±1.36 h) (p<0.001). This is similar to a study of 200 pregnant women by Al Ibraheem et al., in which half of the participants received 25 µg of intravaginal misoprostol every six hours for a maximum of four doses, whereas the other half had an intracervical 16F Foley catheter inserted in addition to misoprostol [12]. The mean induction to delivery time was significantly shorter in the combined intracervical Foley catheter + misoprostol group (15 hours) than in the misoprostol alone group (19 h) (p=0.001).

In our study, the number of vaginal deliveries was slightly higher in Group A at 46 (62.2%) compared to Group B at 45 (60.8%) (p=0.50). This result agreed with a study by Gilani et al. [4], which included 96 pregnant women aged 18-35 years with a singleton cephalic fetus randomized into two groups, Group A (Foley catheter + misoprostol) and Group B (misoprostol alone); vaginal delivery was seen in 36 (75%) women in Group A and 26 (54.17%) women in Group B (p=0.03). In the present study, the mean Bishop’s score was 2.91 ± 0.75 in Group A and 2.83±0.75 in Group B (p=0.515), which was not a statistically significant difference. A study by Eser et al. showed that the mean Bishop’s score was 2.5±0.70 in the group with intravaginal PGE2 alone and 2.7 ± 0.90 in the group with intracervical foleys balloon catheter + vaginal PGE2 [14]. Druenne et al. showed that the mean Bishop’s score was 2.6 +- 0.60 in the group with Foleys catheter + misoprostol and 2.4 +- 0.70 in the group with misoprostol alone [15].

The current study showed a statistically significant difference between both groups in neonatal outcomes and meconium-stained amniotic fluid, which were both higher in Group B than in Group A. Fewer patients had intrapartum complications in Group A (n=1; 1.4%) than in Group B (n=10; 13.5%), which was a statistically significant difference (p=0.0169). In addition, fewer patients had atonic PPH in Group B (n=2; 2.7 %) than in Group A (n=5; 6.8 %), which was not a statistically significant difference (p=0.2453).

Although the randomized design of this study was one of its strengths, it was limited by the inability to blind the interventions. Accordingly, further multicenter trials with a larger sample size are needed to confirm the safety and effectiveness of those methods.

## Conclusions

Our results showed that an intracervical Foley catheter with 25 µg of misoprostol was more effective for IOL than 25 µg of intravaginal misoprostol alone every six hours for a maximum of four doses in terms of shortened induction to delivery interval, meconium-stained amniotic fluid, mode of delivery, and puerperal infection. The intravaginal misoprostol alone group had a slightly decreased vaginal delivery rate and increased cesarean section producing significant complications (e.g., meconium-stained amniotic fluid, intrapartum hemorrhage, intrapartum vomiting, and puerperal infection) than the intracervical Foley catheter and misoprostol group. Mean Bishop’s score was lower in the misoprostol group compared to the intracervical Foley catheter and misoprostol group. Atonic PPH was lower in the misoprostol group than in the intracervical Foley catheter and misoprostol group.
